# Blended Care in Patients With Knee and Hip Osteoarthritis in Physical Therapy: Delphi Study on Needs and Preconditions

**DOI:** 10.2196/43813

**Published:** 2023-07-07

**Authors:** Franziska Weber, Corelien Kloek, Angela Arntz, Christian Grüneberg, Cindy Veenhof

**Affiliations:** 1 Division of Physiotherapy Department of Applied Health Sciences University of Applied Health Sciences Bochum Bochum Germany; 2 Department of Rehabilitation, Physiotherapy Science & Sports University Medical Center Utrecht Utrecht Netherlands; 3 Research Group Innovation of Human Movement Care HU University of Applied Sciences Utrecht Utrecht Netherlands

**Keywords:** telerehabilitation, osteoarthritis, physical therapy, knee, hip, blended, preconditions, Delphi, focus group, user need

## Abstract

**Background:**

Osteoarthritis is a major public health concern. Despite existing evidence-based treatment options, the health care situation remains unsatisfactory. Digital care options, especially when combined with in-person sessions, seem to be promising.

**Objective:**

The aim of this study was to investigate the needs, preconditions, barriers, and facilitators of blended physical therapy for osteoarthritis.

**Methods:**

This Delphi study consisted of interviews, an online questionnaire, and focus groups. Participants were physical therapists, patients with hip and/or knee osteoarthritis with or without experience in digital care, and stakeholders of the health care system. In the first phase, interviews were conducted with patients and physical therapists. The interview guide was based on the Consolidated Framework For Implementation Research. The interviews focused on experiences with digital and blended care. Furthermore, needs, facilitators, and barriers were discussed. In the second phase, an online questionnaire and focus groups served the process to confirm the needs and collect preconditions. The online questionnaire contained statements drawn by the results of the interviews. Patients and physical therapists were invited to complete the questionnaire and participate in one of the three focus groups including (1) patients; (2) physical therapists; and (3) a patient, a physical therapist, and stakeholders from the health care system. The focus groups were used to determine concordance with the results of the interviews and the online questionnaire.

**Results:**

Nine physical therapists, seven patients, and six stakeholders confirmed that an increase of acceptance of the digital care part by physical therapists and patients is crucial. One of the most frequently mentioned facilitators was conducting regular in-person sessions. Physical therapists and patients concluded that blended physical therapy must be tailored to the patients’ needs. Participants of the last focus group stated that the reimbursement of blended physical therapy needs to be clarified.

**Conclusions:**

Most importantly, it is necessary to strengthen the acceptance of patients and physical therapists toward digital care. Overall, for development and usage purposes, it is crucial to take the needs and preconditions into account.

**Trial Registration:**

German Clinical Trials Register DRKS00023386; https://drks.de/search/en/trial/DRKS00023386

## Introduction

Osteoarthritis (OA) is a major public health problem with a high prevalence worldwide, which will further increase in the coming years due to the aging population, rising obesity rate, and people being physically inactive [1]. In particular, the burden of OA on the health care system is expected to grow exponentially [1]. While effective treatment is available, conservative treatment options (especially physical training and education) are still underutilized [2]. Therefore, it is crucial to find effective and efficient treatment strategies to face this challenge.

To facilitate the access to primary care and to reduce health-related costs, digital health care is a promising approach. In particular, when considering the course of the COVID-19 pandemic, the potential of digital health care has been demonstrated, confirming that it is not simply a trend [3]. A general definition of digital health care is the application of information and communication technologies across a broad range of activities performed in health care [4]. Innovations in digital health care enable appropriate and efficient care and offer a range of effective digital health interventions for various somatic problems [5]. Such approaches provide high accessibility at any time and place, may attract people who do not make use of traditional physical therapy services, and are easily scalable [6]. However, the challenge of digital health care is the adherence to the treatment and the missing patient-provider relationship [7]. Linking the advantages of online and offline guidance and treatment yields positive outcomes, since this approach combines the best of two worlds. Integrating in-person and digital health care is referred to as “blended care.” [8]. On the one hand, blended care overcomes the barriers of using solely digital health care, such as low adherence rates to the treatment [7,9,10]. On the other hand, blended care includes the benefit of personal attention of a health care professional. If the digital health focuses on patient empowerment, blended care potentially increases and facilitates a patient’s self-management and ultimately decreases costs [9,11,12]. In the Netherlands, a blended physical therapy intervention called *e-Exercise* has already proven its potential for people with hip or knee OA [13]. This *e-Exercise* intervention revealed the same effectiveness with less physical therapy sessions compared to traditional physical therapy [13].

However, it is important to note that blended care is not suitable in all cases, potentially because of variations in the preferences and motivation of patients, severity of illness, comorbidities, level of education, and digital and health literacy [14,15]. In addition, blended care has to meet the needs of the physical therapists. Thus, to optimize the usage of blended care approaches in an outpatient setting, it is important to involve both patients and physical therapists as well as other relevant stakeholders to take their needs and preconditions into account [16].

Therefore, the objective of this study was to obtain insight on the needs, preconditions, barriers, and facilitators regarding blended physical therapy in patients with knee and hip OA from the perspective of patients, physical therapists, and other stakeholders of the health care system.

## Methods

### Design

**Figure 1:**
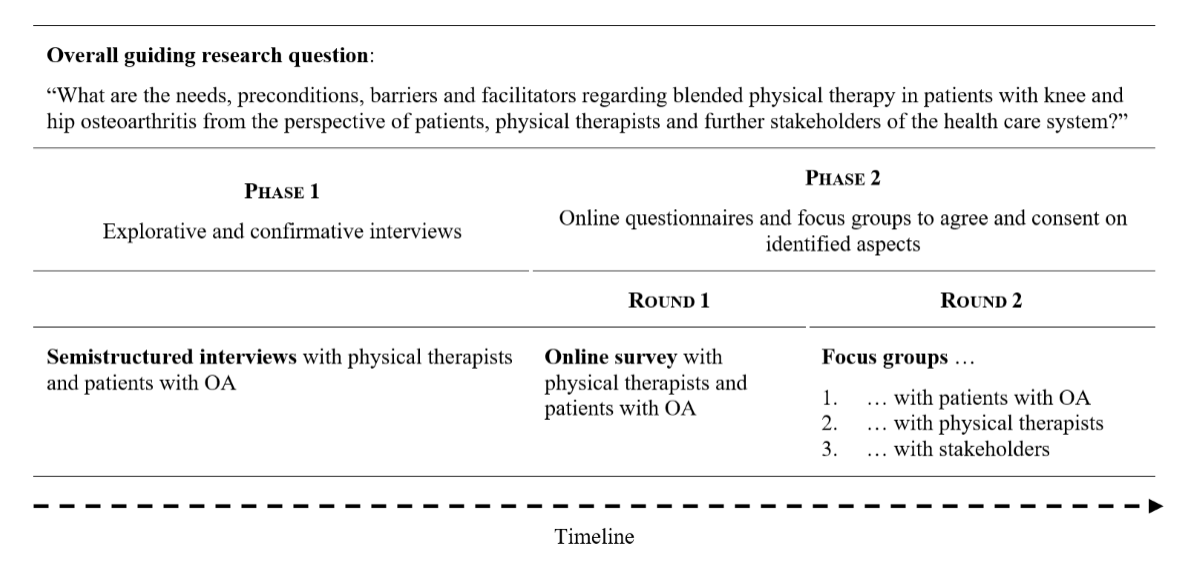
Study method flow chart. OA: osteoarthritis.

A Delphi method was used [17] aiming to obtain insight into the needs, preconditions, facilitators, and barriers with respect to the content, sequence, and ratio of blended physical therapy. Established methodological criteria for reporting Delphi studies were followed to ensure quality [18]. The study design is shown in Figure 1.

### Ethics Considerations

This study was conducted in accordance with the Declaration of Helsinki [19]. The ethics committee of the University of Applied Health Sciences Bochum approved the study (201116_Grüneberg, 04.01.2021). All participants gave written informed consent before data collection began.

### Participants

#### Physical Therapists

We recruited physical therapists using the database of clinical cooperation partners of the University of Applied Health Sciences (Bochum) and through personal networks. To be eligible, physical therapists needed to be registered physical therapists (have a degree in physical therapy) and work in an outpatient physical therapy setting. Furthermore, they needed to have at least 5 years of experience in treating patients with hip or knee OA, give informed consent, be able to understand and speak German, have access to the internet, and own a digital device (eg, tablet, smartphone, or laptop).

#### Patients

Participating physical therapists were asked to contact eligible patients with OA and sent them an information letter regarding the study. Furthermore, patients were recruited through personal networks (eg, via patient associations). Inclusion criteria for the patients were medically diagnosed idiopathic OA of the knee or the hip and signed informed consent. Further criteria were to be able to understand and speak German, have received at least one prescription for physical therapy regarding their OA-related symptoms, own a digital device (eg, tablet, smartphone, or laptop), and have internet access.

The aim was to recruit both physical therapists and patients who already had experience with digital health care in any context, as well as physical therapists and patients who did not have this experience. Participants were recruited until saturation was reached, which was when no new information would be identified from the last two interviews [20]. Theoretical sampling was used [21].

#### Stakeholders of the Health Care System

To obtain a broad distribution of participants, we aimed to recruit a member of a patient association, an owner of a physiotherapeutic practice, a physician, a politician in the field of health care, a person of a health insurance company, a representative of a company developing digital devices, and a member of a physical therapy association. We recruited these stakeholders through patient associations, assisted by a German physical therapy association and through personal networks. To be eligible, participants needed to have at least 5 years of professional experience in their field, internet access, own a digital device (eg, tablet, smartphone, or laptop), give signed informed consent, and have sufficient skills in German.

The inclusion and exclusion criteria were screened via telephone before study participation for all participants.

### Procedure

The Delphi process consisted of two phases; phase 1 included explorative and confirmative interviews and phase 2 included an online questionnaire and focus groups to agree and consent on identified aspects, which was separated in two rounds (Figure 1).

Phase 1 was an explorative phase with the aim to capture different perspectives. Both patients and physical therapists filled out questionnaires regarding demographic data (age, gender, educational level, and experience with digital/blended care) and their (digital) health literacy assessed by the European Health Literacy Survey Questionnaire (HLS-EU-Q16) and the eHealth Literacy Scale (eHEALS) [22,23]. Further, they were asked to participate in individual semistructured interviews via telephone. Two slightly different questionnaires were used for patients and physical therapists, respectively. Topics for the interviews were developed on the basis of the Consolidated Framework For Implementation Research (CFIR) (see the interview guides for patients and physical therapists in Multimedia Appendix 1) [24]. The CFIR consists of the following five domains: (1) characteristics of the individuals involved, (2) innovation characteristics, (3) inner setting, (4) outer setting, and (5) the process of implementation [24]. The process of implementation was not questioned, since there was no specific intervention to implement at that point. Each participant was asked about their experiences with digital health care, and the possible facilitators and barriers they experienced or would expect from digital and blended care in the four domains of the CFIR. In between, a short video [25] was presented during each interview, which showed an example of blended care (combination of in-person physical therapy, video conference, and app) and gave a definition of blended care to create a common understanding. Blended care was defined as an approach in which digital health care is integrated into regular physical therapy.

The aim of phase 2, consisting of two rounds, was to agree and consent on needs, barriers, facilitators, and preconditions for blended care in physical therapy. The same group of physical therapists and patients was invited to fill out an (anonymous) online questionnaire via a secured online platform (SoSci Survey) in round one. Two researchers (AA and FW) translated the results of the interviews in phase 1 into statements; the participants had to agree or to disagree on these statements measured on a 4-point Likert scale from “I completely disagree” (1) to “I completely agree” (4). For instance, if the majority of the participants in the interviews stated that they would like to be taught physical exercises in person, the corresponding statement would be “I prefer the instruction of physical exercises within in-person sessions.” The online questionnaire was quantitatively evaluated and the results were used for round two of this phase. At the beginning of the second round, the results from the questionnaire were briefly presented and the aim of the focus group was explained. The focus groups were conducted via Zoom, version 5.13.5 (12053). Online pin boards (Padlets) were used to present the findings from the online questionnaire and to create a good overview for the participants of the focus groups. The content was structured to individual, innovation, inner setting, and outer setting domains. The focus groups were moderated by one researcher to guide the group through the different topics and come up with specific preconditions for further development and usage of blended care concepts. Three focus groups (patients, physical therapists, and stakeholders) were conducted to agree and consent on results of the online questionnaire (Figure 1). In addition, the aim was to examine what essential preconditions are necessary to make blended physical therapy feasible in an outpatient practice.

### Data Analysis

#### Phase 1

Two researchers (AA and FW) transcribed verbatim and coded the transcripts of the interviews. Data analysis of the interviews was performed based on the framework approach [26]. Using explorative data analysis for each main topic from the interview scheme, citations were extracted and arranged into themes and subthemes. Subsequently, these themes were discussed between the researchers (AA, FW) until consensus was reached; the complete list of themes and subthemes is presented in Multimedia Appendix 2. Finally, all codes of each theme of every participant were displayed in a table [27]. Next, one researcher (FW) examined the raw data again to ensure the robustness of the analytical process and to confirm that all data were indeed reflected in the coding. Transcription, coding, organization, and analysis were performed using MAXQDA Plus 2020, Windows version 20.3.0.

#### Phase 2

Data from round one were exported from the secure online platform into an Excel sheet. Demographics, data from the (digital) health literacy questionnaires, as well as data from the online questionnaire were analyzed descriptively with SPSS (IBM SPSS Statistics 25). Results were analyzed by quantifying scores on each item from the questionnaire and calculating percentages of patients and physical therapists who chose a certain answer on the items.

In round two, focus groups were recorded in writing protocols. Data were categorized into the corresponding themes or subthemes of the interviews according to the CFIR domains. Categorization was discussed between two researchers (CG and FW) until consensus was reached. Data were screened regarding repetitions and each theme and corresponding subthemes were summarized.

## Results

### Participants

Nine physical therapists and seven patients participated in the interviews and the online questionnaire. Five of the physical therapists and four of the patients took part in the focus groups, respectively, and the third focus group consisted of six stakeholders and one physical therapist of the first phase. For physical therapists of phase 1, saturation was reached after nine interviews. The characteristics of physical therapists are shown in Table 1.

Concerning the patients in phase 1, saturation was achieved after seven interviews. Table 2 displays the characteristics of patients. One physical therapist with experience in digital health joined the other stakeholders in the last focus group. The politician in the field of health care was not able to participate in the focus group.

**Table 1:** Characteristics of physical therapists (N=9).

Characteristics		Value
Age (years), mean (SD)		33.0 (6.5)
**Sex, n (%)**		
	Male	4 (44)
	Female	5 (55)
Clinical experience (years), mean (SD)		9.4 (6.2)
Clinical experience in treating patients with OA^a^ (years), mean (SD)		9.4 (6.2)
**Level of education, n (%)**		
	Masters, diploma, state examination (university [of applied sciences]); EQF^b^ Level 7	2 (22)
	Bachelors (university or university of applied sciences); EQF Level 6	6 (67)
	State examination/completion of a vocational training; EQF Level 5	1 (11)
Prior experience in online therapy, n (%)		4 (44)
Working hours/week, mean (SD)		32.0 (12.5)
**General health literacy (HLS-EU-Q16^c^), n (%)**		
	Adequate	4 (44)
	Problematic	4 (44)
	Inadequate	1 (11)
Digital health literacy (G-eHEALS^d^), mean (SD)		32 (7)

^a^OA: osteoarthritis.

^b^EQF: European Qualifications Framework.

^c^HLS-EU-Q16: the European Health Literacy Survey Questionnaire (0=low/no health literacy to 16=high health literacy).

^d^G-eHEALS: German eHealth Literacy Scale (0-40; higher score indicates better digital health literacy).

**Table 2:** Characteristics of patients (N=7).

Characteristics			Value
Age (years), mean (SD)			59.9 (10.8)
**Sex, n (%)**			
	Male	4 (57)	
	Female	3 (43)	
**Osteoarthritis, n (%)**			
	Hip osteoarthritis	3 (43)	
	Knee osteoarthritis	1 (14)	
	Both	3 (43)	
**Time since diagnosis (years), mean (SD)**			
	Hip osteoarthritis	9.8 (8.3)	
	Knee osteoarthritis	7.0 (3.8)	
**Degree of self-reported limitations due to osteoarthritis, n (%)**			
	Fair	2 (29)	
	Mild	5 (71)	
Duration of physical therapy due to osteoarthritis-related symptoms (years), mean (SD)			3.6 (6.0)
**Level of education, n (%)**			
	High	5 (71)	
	Low	2 (29)	
Prior experience in online therapy, n (%)			3 (43)
Adequate general health literacy (HLS-EU-Q16^a^), n (%)			7 (100)
Digital health literacy (G-eHEALS^b^), mean (SD)			32.6 (4.4)

^a^HLS-EU-Q16: European Health Literacy Survey Questionnaire (0=low/no health literacy to 16=high health literacy).

^b^G-eHEALS: German eHealth Literacy Scale (0-40; higher score indicates better digital health literacy).

Textbox 1 summarizes the needs and preconditions of the patients, physical therapists, and the stakeholders regarding blended care, which are the final results of the two phases. The data of the two phases were combined and structured according to the domains of the CFIR.

**Consensus of the needs and preconditions regarding blended physical therapy from the perspective of patients with osteoarthritis, physical therapists, and stakeholders.**

Personal factors (individual)

- Change of the role of physical therapists and gaining new competences
*(Necessity of changing the role of physical therapists, patient and physical therapist being equal partners, new competences are necessary)*
- Attitudes and acceptance (changing attitudes and increasing the acceptance for digital health)
*(Necessity to change attitudes toward and acceptance for digital health)*

Intervention-related factors (innovation)

- Digital content and feature
*(Educational components, information exchange, and exercise program as digital content; important to include motivational strategies in the app such as reminders*
- Usability and operability
*(Easy and intuitive app, necessity of user-friendliness, patient-friendly language, flexibility in decision-making, wide accessibility of the app, feedback through data)*
- Blended care concept (individualization, ratio, and allocation)
*(Individualization is necessary, integration of evidence-based information, regular in-person sessions, 60:40 ratio of online and in-person sessions, flexibility of online or in-person mode*

Organizational factors (inner setting)

- Practice setting (eg, working conditions, personnel structures, hardware)
*(Change of rooms, necessity of hardware, WLAN, software, positive influence on the working conditions, change of personnel structures, change of time schedules, necessity of interoperability of different programs)*
- Change of (interprofessional) cooperation/communication
*(Facilitation of interprofessional communication by online environment)*

System-related factors (outer setting)

- Necessity to change efficiency (eg, time and costs)
*(Time to prepare, efficiency of time, increase in costs, who will pay?)*
- Necessity of clear structural conditions (eg, rules regarding data protection and security)
*(Clear description of concept is necessary, prescription or integration in disease management program is necessary; legal basis; necessity of clear rules and legal aspects regarding data protection; data protection guidelines; implementation of advanced training/ skills)*
- Clear rules and roles before an implementation
*(Development process of digital devices; responsibility for implementation process [stakeholders])*

### Personal Factors (Individual)

#### Change of the Role of Physical Therapists and Gaining New Competences

A changing role of physical therapists was a central precondition for blended care, which received consensus of physical therapists and stakeholders. Different facets of changes have been mentioned; however, the main adjustment was seen in the patient-provider relationship. According to physical therapists, both should be on an equal level with the physical therapist being in a guiding role. There was a full consensus of the physical therapists that blended care has an essential impact to facilitate a patient’s self-management and individual responsibility.

Patients also considered a healthy relationship with and trust in the physical therapist as a crucial precondition for blended care. In contrast to the perspective of physical therapists, passive interventions (and therefore in-person contact) were still one of the most important aspects of physical therapy for patients. Patients were afraid of having less in-person sessions in favor of more digital sessions.

Patients and physical therapists considered adequate communication skills of both groups and a moderate level of health literacy of patients as necessary. From the perspective of physical therapists, a core competence within blended care was the need to be familiar with the technology used. All physical therapists and stakeholders concluded that decision-making is a further competence required if the approach is to be useful and feasible for every patient. As a precondition for using digital health in physical therapy, they mentioned an adequate training of new competences for the physical therapists and gaining positive experiences with digital care for both patients and physical therapists.

#### Attitudes and Acceptance

All participants mentioned the COVID-19 pandemic as a facilitator for blended care, especially increasing the acceptance of digital care. Most of the physical therapists were open regarding digital care. Patients needed and wanted to learn how to handle digital tools in advance. The acceptance of blended care of patients varied; however, in general, they recognized the convenience to exercise anytime and place and incorporating the therapy into their daily lives. Further preconditions to increase the acceptance of patients were the confidence in the physical therapist and sufficient time to learn and practice.

### Intervention-Related Factors (Innovation)

#### Digital Content and Features

The vast majority of all participants considered educational components, information exchange, and an exercise program as content that can be carried out digitally. The results of the online questionnaire regarding the preferred mode of delivery are shown in Table 3.

**Table 3:** Preferred mode of the therapy component (online, in-person, or online and/or in-person) from the perspective of physical therapists and patients (N=16).

Therapy components	Patients (n=6)	Physical therapists (n=9)
First therapy session/getting to know	In-person (n=6)	In-person (n=5)
Information/education session	In-person (n=5)	Online and/or in-person (n=5)
Consultation	Online and/or in-person (n=4)	Online and/or in-person (n=7)
Screening process/diagnostic process	In-person (n=4)	Face-to-face (n=6)
Instruction of exercises	In-person (n=4)	Online and/or in-person (n=5)
Functional integration of movement into activities of daily living	In-person (n=4)	Online and/or in-person (n=8)
Evaluation/last therapy session	In-person (n=6)	Online and/or in-person (n=9)

All physical therapists agreed on the importance to integrate motivational strategies in the technology, such as with activity trackers and reminders (Table 4).

Physical therapists perceived the digital program within blended care as a guiding tool, whereas patients saw digital components only as a supplement to regular in-person sessions. The results of the online questionnaire including specific software features and content are presented in Table 4.

**Table 4:** Preferred content and features of the digital program within a blended physical therapy approach from the patients’ and physical therapists’ perspectives.

Content and features		Patients (n=7), n (%)	Physical therapists (n=9), n (%)
**Content of the digital program**			
	Exercise/training plans that include PA^a^ and exercises	7 (100)	9 (100)
	Therapy/treatment plans that include goal-appropriate exercises and treatment	7 (100)	9 (100)
	Examination/warning of red flags regarding the treatment of patients with OA^b^	6 (86)	9 (100)
	Test/MI^c^ instructions performed by the patient on their own or by the physical therapist with the patient (eg, 6MWT^d^, TUG^e^)	4 (57)	7 (78)
	Communication/exchange with physicians or other professions	4 (57)	5 (56)
	Information on relevant topics for patients with OA	3 (43)	9 (100)
	Patient-reported outcome measures (eg, KOOS^f^, HOOS^g^)	3 (43)	8 (89)
**Features of the digital program**			
	Chat for communication between physical therapists and patients	6 (86)	7 (78)
	Documentation system for the physical therapist	5 (71)	7 (78)
	Agenda with future physical therapy appointments	5 (71)	3 (33)
	Video chat	4 (57)	9 (100)
	Diary of patients to collect PA and exercises	4 (57)	8 (89)
	Collecting/capturing of data of the course of therapy of the patient	4 (57)	7 (78)
	Reminder messages of appointments	3 (43)	9 (100)
	Reminder messages of PA	2 (29)	8 (89)

^a^PA: physical activity.

^b^OA: osteoarthritis.

^c^MI: measurement instrument.

^d^6MWT: 6-minute walking test.

^e^TUG: timed “up & go” test.

^f^KOOS: Knee Injury and Osteoarthritis Outcome Score.

^g^HOOS: Hip Disability and Osteoarthritis Outcome Score.

#### Usability and Operability

Patients and physical therapists had the same opinion regarding the importance of technology being user-friendly. From their perspective, digital tools should be easy and intuitive to use.

#### Blended Care Concept

All participants agreed that blended care must be tailored to the patients’ individual needs. Participants considered in the online questionnaire an average ratio of 60/40 digital/in-person sessions as optimal (Table 5). Physical therapists and patients considered that a first in-person session is crucial, and that the longer the treatment process, the less in-person sessions are necessary. Stakeholders stated that the needs of the patient, access to devices, state of condition and confidence in physical therapy, motivation of the patient, as well as a high level of patients’ self-management are factors that influence the decision on the most appropriate therapy mode.

An academic education and several years of professional experience as a physical therapist were mentioned as preconditions, since this supports the decision on the therapy mode from the perspective of the stakeholders.

The stakeholders emphasized the value of “taking the physical therapist home,” which would increase the sustainability of therapy in their point of view.

**Table 5:** Preferred ratio of online and in-person therapy of patients with osteoarthritis.

Online/in-person ratio	Patients (n=7)	Physical therapists (n=9)
0%/100%	1	0
10%/90%	0	0
20%/80%	1	0
30%/70%	1	0
40%/60%	1	0
50%/50%	1	2
60%/40%	1	3
70%/30%	1	4
80%/20%	0	0
90%/10%	0	0
100%/0%	0	0

### Organizational Factors (Inner Setting)

#### Practice Setting

Patients and physical therapists considered a separate room only for digital care (eg, video conference) as necessary to be undisturbed, maintain privacy of the patient, and having all equipment ready to use.

Physical therapists considered a change of practice structures as necessary. Proper time planning is important (eg, to prepare digital sessions). The stated preconditions regarding a practice setting for the usage of blended care are summarized in Table S1 of Multimedia Appendix 3.

A precondition for blended care was that every user has access to digital devices and a stable internet connection. Physical therapists preferred tablets or laptops as hardware. Patients considered missing equipment and technical requirements as a barrier for blended care. They preferred a large screen on their digital devices. The stakeholders stated the importance of the interoperability of different systems, especially with already existing systems.

#### Change of (Interprofessional) Cooperation/Communication

The interviewed physical therapists expected a facilitation and simplification of the interprofessional communication and cooperation within blended care. For instance, data should be collected and stored in a more structured way and the treating physician would have the option to access the status or progress of the patient; in that way, the communication between the physical therapist and physician will be based on results and data. Further, the transfer of a patient to another physical therapist can be easily achieved.

### System-Related Factors (Outer Setting)

#### Necessity to Change Efficiency

Stakeholders concluded that time is an advantage but also a disadvantage. For instance, blended care could save time when filling out questionnaires in advance; however, there is more time needed for preparation. All participants were in accordance that the financial reimbursement for blended care needed to be clarified (eg, time for preparation and for the digital care part, costs for licenses and systems). Stakeholders determined that health insurance companies needed to cover the costs for in-person and digital care. Therefore, the single blended care intervention needed to be specified and described well.

#### Necessity of Clear Structural Conditions

Structural preconditions mentioned included legal requirements, proof of effectiveness, data protection, and security. Stakeholders suggested certifications for each type of technology, which meet data protection guidelines. Additionally, physical therapists suggested educating patients regarding data protection and security.

An (advanced) training for physical therapists should particularly focus on digital communication, data protection issues, and evidence-based digital care. Patients should particularly be educated regarding the handling of technology.

#### Clear Rules and Roles Before Implementation

Stakeholders concluded that important steps before an implementation of blended care are its communication and promotion, dealing with resistance, training of physical therapists as specialists, and well-prepared introduction of technologies.

Structural facilitators were seen in the COVID-19 pandemic and if patients were provided with digital devices. The competitive market, missing transparency, privacy issues, and different understandings of blended care were considered as structural barriers. All facilitators and barriers regarding blended physical therapy are listed in Figure S1 of Multimedia Appendix 3.

## Discussion

This study investigated different perspectives of patients, physical therapists, and stakeholders on blended physical therapy of patients with OA.

Overall, patients and physical therapists are skeptical about blended physical therapy, which can be seen in the results of both groups. For instance, there was low patient acceptance of the digital care part; patients and physical therapists expressed the importance of in-person care and the integration of in-person treatment at the beginning and the end of each therapy session. They were afraid that the digital care part could replace the in-person sessions with their therapist, which are crucial for them. Thus, blended physical therapy is currently unknown for both patients and physical therapists. Since it will fit into future care models, it is still crucial to acquaint patients and physical therapists with blended physical therapy. Therefore, it is important to listen carefully to the preconditions, facilitators, and barriers raised by both the patients and physical therapists.

The most commonly stated facilitators of blended physical therapy according to all participants were the individualization of blended physical therapy, the user-friendliness of the technology, the COVID-19 pandemic, access to digital devices, and a stable internet connection. Barriers of blended physical therapy included technical skills of patients and physical therapists, costs, as well as society’s lack of knowledge and information regarding blended physical therapy interventions.

One major finding was that the acceptance of the digital care part within blended physical therapy is still quite low in patients, whereas physical therapists are more open to using this technology. Interestingly, the Dutch *e-Exercise* project revealed a reverse trend in this regard, in which patients were more enthusiastic and physical therapists more critical [9]. This is quite remarkable, since it is most likely due to the fact that the patients had experiences with a specific blended intervention, which clearly influenced their opinion and attitude toward blended physical therapy. Therefore, it seems crucial to gain positive experiences with blended physical therapy [28]. In contrast, physical therapists had mixed experiences with *e-Exercise*, since the workload increased and it was more time-consuming, especially at the beginning [29]. Patients, who did not have any experience with digital care, were more skeptical and expected more barriers to its use. A further personal precondition that was raised was the learning of new competences. Patients, as well as physical therapists, seem to be open and willing to learn new competences, which can possibly increase the acceptance and change their attitudes regarding blended physical therapy [30,31]. This has also been mentioned in previous studies as a key facilitator for the uptake and acceptance of digital care [30,31].

An intervention-related precondition is to have a first and last in-person physical therapy session. This aspect was crucial for physical therapists, since they have difficulties imagining performing a thorough first assessment or evaluation digitally [28,32].

A further intervention-related precondition is the individualization of care. A key finding was that there is no “one-size-fits-all” solution, but rather there is a necessity to tailor blended physical therapy to the specific needs of each patient. This is mentioned as a main advantage of blended physical therapy, since it is beyond the borders of traditional care to provide, for instance, immediate and automated feedback specifically tailored to the patient [11,28,30]. While they still have the opportunity to see their patient in person, they will also have more time for other interactions such as in-depth conversations and personal attention. In general, physical therapists need to have the possibility to act flexibly and to have the competence to decide whether or not a patient is suitable for blended care. The Dutch *Blended Physiotherapy Checklist* already supports and guides physical therapists in their clinical reasoning process while setting up a personalized blended physical therapy intervention [14].

Important preconditions regarding organizational factors are the interoperability of different types of software. In particular, the physical therapists need to use different systems (eg, administration, training programs), which is a deterrent to use without data transfer between the systems [33]. Therefore, information technology companies have the responsibility to develop interfaces between systems to enable interoperability. A change of facilities is also necessary to create sufficient privacy and a safe space for the physical therapist and the patient (eg, while having a video conference) [28,34].

The main system-related precondition is the reimbursement of blended physical therapy, which is also an issue in different countries [15,34-36]. Even though the COVID-19 pandemic enabled reimbursement of telehealth services, there is still no permanent solution [35]. Since there is still a lack of a payment solution, it is recommended to conduct pilot studies to investigate the usability and effectiveness of specific blended physical therapy approaches keeping the mentioned preconditions, facilitators, and barriers identified in this study in mind. Furthermore, it is important to obtain a clear picture of data protection and safety issues. Stakeholders consented to have certificates for software, which help to obtain an overview as a user and rates technologies regarding their value, which is already in place in some countries [15,34]. Independent, public institutions might generate these guidelines, certificates, and overviews for users. A further important system-related precondition raised was the development of an advanced training program for digital competences, which can be integrated in the curriculum of undergraduate and postgraduate physical therapist training programs. Therefore, it is necessary to create a framework of digital competences [37].

An important strength of this study is the investigation of blended physical therapy and not solely digital care. Simultaneously, it is challenging to investigate these two concepts separately, since they are very connected and participants had difficulties in distinguishing between them. Therefore, parts of the results relate to digital care in general and not solely to blended physical therapy. A further strength is the inclusion of both the patient and the physical therapist perspectives, which is complemented by a final discussion of stakeholders. Additionally, the recruitment of two different groups of patients and physical therapists (with and without experience in digital health) contributed to a holistic picture. Limitations of our study are that our findings cannot be generalized to every type of blended physical therapy, since they may differ. In particular, showing the video with an example of blended care to the participants affected the results. It could be possible that needs, barriers, facilitators, and preconditions would vary if a completely different blended care concept would be introduced. Furthermore, two researchers held the interviews, which might have influenced the flow of the interviews in different ways. To prevent this, a topic guide was used, which supported covering the main topics.

Although both patients and physical therapists were not too enthusiastic about blended physical therapy, consensus on the needs and preconditions of blended physical therapy serves as a principal foundation for relevant caregivers, stakeholders, and researchers. Needs, preconditions, facilitators, and barriers have been indicated in four domains. The findings underline the importance of developing blended physical therapy interventions with a whole group of different stakeholders, which is crucial to facilitate the use and implementation of blended physical therapy at a later stage.

## Appendix

Multimedia Appendix 1 Semistructured interview guides for the interviews with (1) patients and (2) physical therapists.

Multimedia Appendix 2 List of themes and subthemes of the data analysis of the interviews.

Multimedia Appendix 3 Table S1 and Figure S1.

## Abbreviations

CFIR: Consolidated Framework For Implementation Research
eHEALS: eHealth Literacy Scale
HLS-EU-Q16: European Health Literacy Survey Questionnaire
OA: osteoarthritis

## Data Availability

Data are available on reasonable request. Data are in the form of digital voice recording of interviews, which were also transcribed verbatim into Word files. Data of the online questionnaire and demographics are available as Excel files. Data of the focus groups are available in form of written protocols as Word files. These data are stored in a password-secured research drive, which is only accessible to author FW. Voice recordings contain identifiable data, which will not be available on request to maintain anonymity of the participants. The other files with deidentified participant data may be made available on reasonable request.
