# Supplementary material for: Blended Care in Patients With Knee and Hip Osteoarthritis in Physical Therapy: Delphi Study on Needs and Preconditions
Source: JMIR Rehabil Assist Technol. 2023 Jul 7;10:e43813. doi: 10.2196/43813 (PMC10362426; doi:10.2196/43813)
Supplement: Multimedia Appendix 1 [file rehab_v10i1e43813_app1.pdf]

Multimedia Appendix 1: Semistructured interview guides for the interviews with (1) the patients and with (2) the physical therapists

1. Interview guide for patients

Guide for patients **who have experience with digital (health) care** and patients who have no experience with digital (health) care and questions for both groups.

Research question:

"What are the needs, preconditions, barriers and facilitators regarding blended physiotherapy in patients with knee and/or hip osteoarthritis from the perspective of patients, physiotherapists and further stakeholders of the health care system?"

Introduction (not recorded)

- Greetings and appreciation for taking time
- Brief description of the interview procedure and approximate duration, information on recording equipment
- Emphasis on the voluntary nature of the participation and the possibility of stopping the interview at any time
- Encouraging open and free narration, no evaluation of what is said and no right or wrong
- Clarification of questions

No	Interview question	Questions to keep up the narrative flow	Operationalization
**Previous experience with physical therapy regarding osteoarthritis**			
1	What were the main aspects of your previous physiotherapeutic care regarding your osteoarthritis related symptoms?	• For instance, aspects are examination, information/education, exercises, physical activities and evaluation.	Characterization of the participants
2	What were important aspects of your previous physiotherapeutic care regarding your osteoarthritis related symptoms?	• Think about the aspects just mentioned and name those, which you consider as important.	Characterization of the participants
**Use and interaction with digital (health) applications**			
3	**How do you use digital (health) applications in your daily life?**<br>***(Definition of a digital (health) application)***	• **Which digital (health) application do you use?**<br>• **For which purpose do you use digital (health) applications?**<br>• **Which device (PC, laptop, tablet, smartphone) do you use for your digital (health) applications?**<br>• **How often do you use digital (health) applications?**	Characterization of the participants

		• **To what extent has the use of digital (health) applications in your daily life changed due to the Covid-19 pandemic?**	
**4**	Why are you not using digital (health) applications in your daily life?<br>*(Definition of a digital (health) application)*	• What is the reason for the non-usage?<br>• Please describe it in more detail.	Characterization of the participants
**5**	How would you rate your own skills in using digital (health) applications?	• Please specify.<br>• Please give examples.	Characterization of the participants
**Use of digital (health) care in general**			
**6**	What is your opinion on the use of digital (health) applications in health care?<br>What are the advantages and disadvantages?	• To which extent has, the Covid-19 pandemic changed your attitude towards digital (health) care.<br>• Please describe it in more detail.<br>• Please give an example to illustrate it in more detail.	Individual
**7**	What experience did you make within the health care sector regarding the use of digital (health) care?	• How did you experience it?<br>• Please give an example, so I can easily imagine, what you mean.	Individual
**Use of digital (health) care in physiotherapy**			
**8**	How do you imagine the ideal physiotherapeutic care in 10 years?	• Does your imagination change when you think of the possibilities of digitalization?<br>• Do you have any other suggestions?	Individual
**In between: presentation of a video as an example of a blended care situation: "Now, an explanatory video follows, providing an example of how a blended care approach can be performed in practice. At the end of the video, a written definition of blended care is given."**			
**9**	What do you think about it, when you see this example of a blended care intervention?	• What are your first thoughts regarding this example?	Individual
**10**	What are barriers to implement blended care in physical therapy?	• Please describe it in more detail.<br>• Can you think of anything else that comes to your mind?	Individual
**11**	What are facilitators to implement blended care in physical therapy?	• Please describe it in more detail.<br>• Can you think of anything else that comes to your mind?	Individual
**12**	How would blended care change the physical therapist-patient-relationship?	• Can you think of anything else that comes to your mind?	Inner Setting

		• Please describe it in more detail.	
**12**	Which skills/knowledge should you provide as a patient in order to implement blended care?	• Can you think of anything else that comes to your mind?<br>• Please describe it in more detail.	Inner Setting
**13**	Which conditions would have to be fulfilled to make blended care feasible in physical therapy?	• Think of technical, practical, personnel, financial and organisational (pre-) conditions.<br>• Can you think of anything else you would like to add?	Outer Setting
**14**	What are your needs for the content of digital (health) applications, embedded in a blended care approach regarding osteoarthritis?	• You mentioned earlier, that …. What do you mean with that?<br>• Can you think of anything else that comes to your mind?<br>• Please describe it in more detail.	Intervention
**15**	What treatment aspects/parts (examination, information/education, exercises, physical activities, evaluation) do you think would be suitable for online or in-person therapy?	• Which medium (video, video chat, app, pc program …) would be best suited for which aspect/part?<br>• Which aspect/part of the physiotherapeutic treatment should be delivered via which digital device (tablet, smartphone, pc, laptop …)?<br>• Why?	Intervention
**16**	What is the ideal percentage distribution of online or personal contact over the entire process of physical therapy for you?	• Please explain your opinion.	Intervention
**17**	Do you have any further comments on this topic?	• Are there any further questions?<br>• Is there anything, you would like to highlight?	Closure of the interview

Finishing the interview (not recorded)

- Appreciation for the cooperation and small gift
- Note: contact details of the university and contact person for any open/further questions or for clarifying discussions

Definition: Digital (health) application

Digital (health) applications are programs/software that fulfil certain functions and are offered digitally (via smartphone, tablet, pc or laptop). Such as an app, that counts your steps.

2. Interview guide for physical therapists

Guide for physical therapists **who have experience with digital (health) care** and physiotherapists <u>who have no experience with digital (health) care</u> and questions for both groups.

Research question:

"What are the needs, preconditions, barriers and facilitators regarding blended physical therapy in patients with knee and/or hip osteoarthritis from the perspective of patients, physical therapists and further stakeholders of the health care system?"

Introduction (not recorded)

- Greetings and appreciation for taking time
- Brief description of the interview procedure and approximate duration, information on recording equipment
- Emphasis on the voluntary nature of the participation and the possibility of stopping the interview at any time
- Encouraging open and free narration, no evaluation of what is said and no right or wrong
- Clarification of questions

No	Interview question	Questions to keep up the narrative flow	Operationalization
Previous experience with physiotherapeutic care of people with osteoarthritis			
1	What are the **different aspects** regarding your physiotherapeutic care of patients with osteoarthritis?	• For instance, aspects are examination, information/education, exercises, physical activities and evaluation.	Characterization of the participants
2	What are **important aspects** regarding your physiotherapeutic care of people with osteoarthritis in your opinion?	• Think about the aspects just mentioned and name those, which you consider as important.	Characterization of the participants
3	To which extent does the treatment vary in relation to the status of osteoarthritis of your patients?	• Please explain.	Characterization of the participants
Use and interaction with digital (health) applications			
4	**How do you use digital (health) applications in your daily life?**	• **Which digital (health) application do you use?**	Characterization of the participants

	(Definition of a digital (health) application)	• **For which purpose do you use digital (health) applications?**<br>• **Which device (PC, laptop, tablet, smartphone) do you use for your digital (health) applications?**<br>• **How often do you use digital (health) applications?**<br>• **To what extent has the use of digital (health) applications in your daily life changed due to the Covid-19 pandemic?**	
5	Why are you not using digital (health) applications in your daily life?<br>*(Definition of a digital (health) application)*	• What is the reason for the non-usage?<br>• Please describe it in more detail.	Characterization of the participants
6	How would you rate your own skills in using digital (health) applications?	• Please specify.<br>• Please give examples.	Characterization of the participants
Use of digital (health) care in general			
7	What is your opinion on the use of digital (health) applications in health care?<br>What are the advantages and disadvantages?	• To which extent has, the Covid-19 pandemic changed your attitude towards digital (health) care.<br>• Please describe it in more detail.<br>• Please give an example to illustrate it in more detail.	Individual
8	What experience did you make within the health care sector regarding the use of digital (health) care?	• How did you experience it?<br>• Please give an example, so I can easily imagine, what you mean.	Individual
Use of digital (health) applications in physical therapy			
9	How do you imagine the ideal physiotherapeutic care in 10 years?	• Does your imagination change when you think of the possibilities of digitalization?	Individual

		• Do you have any other suggestions?	
In between: presentation of a video as an example of a blended care situation: "Now, an explanatory video follows, providing an example of how a blended care approach can be performed in practice. At the end of the video, a written definition of blended care is given."			
9	What do you think about it, when you see this example of a blended care intervention?	• What are your first thoughts regarding this example?	Individual
10	What are barriers to implement blended care in physical therapy?	• Please describe it in more detail.<br>• Can you think of anything else that comes to your mind?	Individual
11	What are facilitators to implement blended care in physical therapy?	• Please describe it in more detail.<br>• Can you think of anything else that comes to your mind?	Individual
12	To which extent would blended care change the physical therapist-patient-relationship?	• Can you think of anything else that comes to your mind?<br>• Please describe it in more detail.	Inner Setting
13	Which skills should you provide as a physical therapist in order to implement blended care?	• Can you think of anything else that comes to your mind?<br>• Please describe it in more detail.	Inner Setting
14	Which conditions would have to be fulfilled to make blended care feasible in physical therapy?	• Think of technical, practical, personnel, financial and organisational (pre-) conditions.<br>• Can you think of anything else, you would like to add?	Outer Setting
15	What are your needs for the content of digital (health) applications, embedded in a blended care approach regarding osteoarthritis?	• You mentioned earlier, that …. What do you mean with that?<br>• Can you think of anything else that comes to your mind?<br>• Please describe it in more detail.	Intervention

16	What treatment aspects/parts (examination, information/education, exercises, physical activities, evaluation) do you think would be suitable for online or in-person therapy?	• Which **medium** (video, video chat, app, pc program …) would be best suited for which aspect/part?<br>• Which aspect/part of the physiotherapeutic treatment should be delivered via which **digital device** (tablet, smartphone, pc, laptop …)?<br>• Why?	Intervention
17	What is the ideal percentage distribution of online or personal contact over the entire process of physical therapy for you?	• Please explain your opinion.	Intervention
18	Do you have any further comments on this topic?	• Are there any further questions?<br>• Is there anything, you would like to highlight?	Closure of the interview

Finishing the interview (not recorded)

- Appreciation for the cooperation and small gift
- Note: contact details of the university and contact person for any open/further questions or for clarifying discussions

Definition: Digital (health) application

Digital (health) applications are programs/software that fulfil certain functions and are offered digitally (via smartphone, tablet, pc or laptop). Such as an app, that counts your steps.

Literature

Interview guide: clustered according to the "Consolidated Framework for Implementation Research (CFIR) from Davies et al., 2020 [1]

1. Davies F, Shepherd HL, Beatty L, Clark B, Butow P, Shaw J. Implementing Web-Based Therapy in Routine Mental Health Care: Systematic Review of Health Professionals' Perspectives. Review. *Journal of medical Internet research*. 2020;22(7):e17362. doi:10.2196/17362
