# Supplementary material for: Blended Care in Patients With Knee and Hip Osteoarthritis in Physical Therapy: Delphi Study on Needs and Preconditions
Source: JMIR Rehabil Assist Technol. 2023 Jul 7;10:e43813. doi: 10.2196/43813 (PMC10362426; doi:10.2196/43813)
Supplement: Multimedia Appendix 2 [file rehab_v10i1e43813_app2.pdf]

Multimedia Appendix 2: List of themes and subthemes of the data analysis of the interviews

List of Themes	Memo
Themes and subthemes	
**Implementation process**	
**Patient-related factors**	This code is used when it concerns the factors, which influence the use/usage of blended care.
Role concept	This code is used when talking about how the patient understands his or her role and the physiotherapists role in the treatment process and what influence the blended care concept has on this understanding of the role and what preconditions must be fulfilled for this change to take place.
Relationship	This code is used when talking about the changes in the relationship between the physiotherapist and the patient from the patient's point of view due to the use of blended care and when stating which conditions must be fulfilled so the relationship does not change negatively despite the use of blended care.
Competences	This code is used to indicate which competences the patient needs to have at a personal level in order to use blended care.
**Acceptance**	
Role of physical therapy	
Digital tools	
**System-related factors**	
Time	This code is used when considering whether the implementation of blended care gives the user more or less time for the treatment process.
Costs/ expenses	This code is used to identify the costs of implementing blended care and who or what system is responsible for covering the costs. Furthermore, it is also included if it is a precondition for the implementation of blended care that the costs are covered.
Data security and patient rights	This code is used if subjects talk about privacy within blended care and if factors are named that require the patient's consent.
Structural (pre-)conditions/ framework conditions	Code is used when talking about changes in work, therapy and acceptance of digital media due to the pandemic and when talking about preconditions for use and structural preconditions for implementing blended care at a system level.
**Intervention-related factors**	
Digital elements	

Quality	This code is used when talking about factors that influence the implementation of blended care and when subjects describe how the quality of care changes because of blended care.
Allocation of the competences	
Order	
**Technical factors**	This code is used when it generally concerns technical requirements for implementation of blended care.
Software	This code is used when talking about formats (video, text form, video chat, etc.), which content should be offered, the functional structure of the online program and which functions it should have. It is also used when talking about the need for WLAN access in order to be able to use blended care.
Hardware	This code is used when it comes to access to digital devices and when it comes to the allocation of digital components to the end devices.
Usability	This code is used when it comes to the requirements that must be fulfilled in order to use digital tools within the blended care intervention and to simplify the usability of the technology.
**Organisational factors**	
Practical setting	
Cooperation/ collaboration	
Working conditions	
Personal structure	
**Therapist-related factors**	
Treatment approach	This code is used when the participant talks about, how he/she is currently treating his/her patients and when he/she talks about how his/her therapy is changing due to the use of blended care.
Relationship	This code is used when talking about changes in the relationship between therapists and patients from the therapist's point of view due to the use of blended care and when stating which conditions have to be fulfilled so that the relationship does not change negatively despite the use of blended care.
Competences	This code is used when talking about which competences the therapist requires on a personal level in order to apply blended care.
