# Supplementary material for: Blended Care in Patients With Knee and Hip Osteoarthritis in Physical Therapy: Delphi Study on Needs and Preconditions
Source: JMIR Rehabil Assist Technol. 2023 Jul 7;10:e43813. doi: 10.2196/43813 (PMC10362426; doi:10.2196/43813)
Supplement: Multimedia Appendix 3 [file rehab_v10i1e43813_app3.pdf]

## Multimedia Appendix 3

Table S1 Checklist of practice setting regarding blended physical therapy in the perspective of patients and physical therapists

| **Practice setting checklist** | |
|---|---|
| Patients | Physical therapists |
| Separate rooms (enough space, calm, privacy) | Facilities (enough space, privacy) |
| Home office physiotherapists (privacy) | Possibility and preparation of home office for physical therapists |
| Equipment must be available | |
| Technology (devices and WLAN) | Technology (devices and WLAN) |
| | Data protection |
| | Concepts should be clear in advance |
| | Proper time schedule |

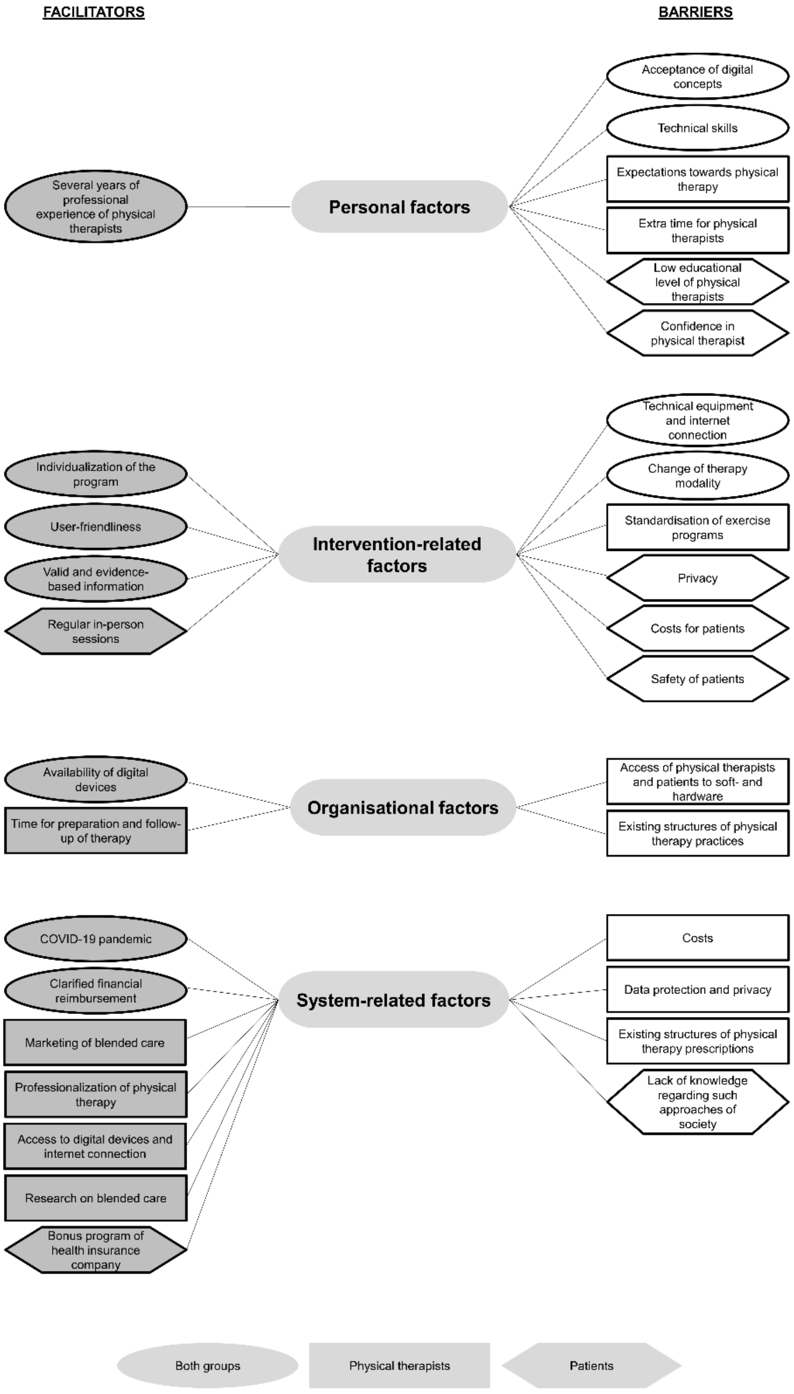


*Figure S1 Facilitators and barriers of blended physical therapy*
